# Hemophagocytic Lymphohistiocytosis Gene Variants in Multisystem Inflammatory Syndrome in Children

**DOI:** 10.3390/biology11030417

**Published:** 2022-03-09

**Authors:** Anshul Vagrecha, Mingce Zhang, Suchitra Acharya, Shannon Lozinsky, Aaron Singer, Chana Levine, Maha Al-Ghafry, Carolyn Fein Levy, Randy Q. Cron

**Affiliations:** 1 Department of Pediatrics, Division of Hematology/Oncology and Cellular Therapy, Cohen Children’s Medical Center, New Hyde Park, NY 11040, USA; sacharya@northwell.edu (S.A.); slozinsky@northwell.edu (S.L.); clevine6@northwell.edu (C.L.); malghafry@northwell.edu (M.A.-G.); clevy4@northwell.edu (C.F.L.); 2 Zucker School of Medicine at Hofstra/Northwell, Hempstead, NY 11549, USA; 3 Division of Pediatric Rheumatology, Children’s of Alabama, University of Alabama at Birmingham, Birmingham, AL 35233, USA; mzhang@peds.uab.edu (M.Z.); randycron@uabmc.edu (R.Q.C.); 4 Yeshiva College, Yeshiva University, New York, NY 10033, USA; alsinger@mail.yu.edu

**Keywords:** MIS-C, HLH, VUS, DOCK8

## Abstract

**Simple Summary:**

Children with a COVID-19 infection are at risk of developing a novel syndrome called multisystem inflammatory syndrome in children (MIS-C). This disease state is characterized by a high level of inflammation. It is unclear why only some children infected with SARS-CoV-2 later develop MIS-C. There may be genetic risk factors for MIS-C development, but none have previously been reported. We report genetic findings in a group of children with MIS-C.

**Abstract:**

Multisystem inflammatory syndrome in children (MIS-C) affects few children previously infected with severe acute respiratory syndrome coronavirus 2 (SARS-CoV-2). In 2020, 45 children admitted to our hospital for MIS-C underwent genetic screening with a commercial 109-immune-gene panel. Thirty-nine children were diagnosed with MIS-C, and 25.4% of the 39 MIS-C patients harbored rare heterozygous missense mutations either in primary hemophagocytic lymphohistiocytosis (pHLH) genes (LYST, STXBP2, PRF1, UNC13D, AP3B1) or the HLH-associated gene DOCK8 (four variants). We demonstrate that foamy virus introduction of cDNA for the four DOCK8 variants into human NK-92 natural killer (NK) cells led to decreased CD107a expression (degranulation) and decreased NK cell lytic function in vitro for each variant. Heterozygous carriers of missense mutations in pHLH genes and DOCK8 may serve as risk factors for development of MIS-C among children previously infected with SARS-CoV-2.

## 1. Introduction

Multisystem inflammatory syndrome in children (MIS-C) is a rare, delayed (~3–8 weeks postinfection) complication of the severe acute respiratory syndrome coronavirus 2 (SARS-CoV-2) infection in children. According to the United States Centers for Disease Control and Prevention (CDC), it is diagnosed in individuals < 21 years presenting with fever, multiorgan dysfunction (>2 organ systems), marked elevation in inflammatory biomarkers, and current or recent evidence of SARS-CoV-2 infection without other known etiologies [1]. Children with MIS-C share clinical features with other inflammatory syndromes, such as primary hemophagocytic lymphohistiocytosis (pHLH), cytokine storm syndrome (CSS), and sepsis [2,3,4,5]. While MIS-C is distinct from these clinical entities, there could be some potential overlap between the underlying immunologic signature of MIS-C and the other conditions on this spectrum of hyperinflammatory disorders. For example, a recent report identified elevated CSS cytokines such as IL-6 and IL-18 in children with MIS-C among other makers of inflammation [5]. Despite these similarities, the pathophysiology of MIS-C is not well-understood.

Genetic predispositions in inflammatory conditions such as HLH have been described. A recent report used computational algorithms to demonstrate that pHLH genes may potentially interact with SARS-CoV-2, predisposing patients to thrombotic complications of severe COVID-19 infection through the release of neutrophilic extracellular traps [6]. Another report suggested adults with germline variants in two pHLH genes (UNC13D and AP3B1) experience increased mortality and severe COVID-19-related CSS [7]. Recently, heterozygous mutations in pHLH genes have been reported in children with secondary forms of HLH/CSS [8,9]. Genetic risk factors for MIS-C remain poorly understood. Given that clinical presentations of MIS-C mimic HLH and other immune dysregulation syndromes, the goal of this study was to investigate genetic variations in key genes crucial to immune regulation among children with MIS-C. Identification of genetic variants among children with MIS-C may help to identify those at risk for MIS-C development following SARS-CoV-2 infection and may provide a better understanding of the disease pathophysiology of MIS-C and related postinfectious hyperinflammatory states.

## 2. Materials and Methods

### 2.1. Identification of Gene Variants in the Patient Cohort

Northwell IRB approved this single-institution retrospective chart review study. Patients admitted with suspected MIS-C from April to July 2020 were included in the analysis, and patients who met the CDC definition of MIS-C were separated into a cohort. As part of the follow up of patients, an expanded genetic panel (monogenic autoimmunity panel) consisting of 109 genes associated with immune dysfunction, including primary HLH genes, was ordered on every patient. (Supplementary Table S1).

Peripheral blood was collected during the first outpatient visit after hospital discharge and sent to a commercial laboratory (Invitae, San Francisco, CA, USA). The resulting genetic variants were classified using guidelines from the American College of Medical Genetics and Genomics. Reported population frequencies were derived from public databases.

Finally, under an IRB-approved protocol, the effect of the DOCK8 gene variants identified in patients was studied in vitro. Human NK-92 natural killer (NK) cells were transduced with human foamy virus (FV) expressing DOCK8 cDNA, representing the various patient-derived DOCK8 missense mutations, to study the effects on NK cell function. The DOCK8 variants were selected since it was the most prevalent gene observed in the patient cohort.

### 2.2. DOCK8 WT and Mutant Gene Preparations

Human DOCK8 expression cDNA was cloned from healthy donor PBMCs from mRNA using reverse transcription (ThermoFisher, Waltham, MA, USA). Sanger sequence analysis confirmed the cDNA sequence was equal to DOCK8 mRNA variant 1 (NM_203447). A recombinant FV expression system required for introduction of large cDNA constructs was kindly provided by Dr. Grant Trobridge at Washington State University (Pullman, WA, USA) [10]. This system contains the pFV-SGW plasmid that expresses the exogenous gene plus enhanced green fluorescent protein (EGFP) driven by the SFFV promoter, as well as three helper plasmids that separately produce gag, pol, and env proteins used for the assembly of recombinant viral particles. The wild-type (WT) DOCK8 cDNA was inserted into pFV-SGW to generate the pFV-SGW-DOCK8-WT plasmid. Based on the WT plasmid, the precise MIS-C patient-derived mutant DOCK8-sequence-equivalent cDNAs were generated by using a QuikChange Lightning Site-Directed Mutagenesis Kit according to the kit instructions (Agilent Technologies, Santa Clara, CA, USA). Each mutant cDNA was confirmed by Sanger DNA sequence analysis.

### 2.3. Recombinant FV Preparation and Infection of NK-92 Cells

WT or mutant DOCK8 pFV-SGWs plus the 3 other assistant plasmids were transfected into exponentially growing HK293T cells by using FuGene HD (Promega, Madison, WI, USA) according to instructions. The supernatants were harvested at 72 h after transfection, followed by supernatant concentration with lenti-X concentrator (Takara, Kusatsu, Japan) according to manufacturer’s recommendations. The concentrated viruses were stored at −70 °C before use. NK-92 cells (1 × 106) were infected by mixing with viruses and incubated overnight in 5% CO_2_ at 37 °C. Infectious status was monitored, and positively infected NK-92 cells were sorted by flow cytometry based on EGFP expression.

### 2.4. NK-92 Cell CD107a Expression Assays (Degranulation Assays)

FV-infected NK-92 cells and K562 erythroleukemia target cells (generously provided by Dr. Olaf Kutch, Department of Microbiology, University of Alabama at Birmingham, Birmingham, AL, USA) were mixed together in a 2:1 ratio and incubated in 5% CO_2_ at 37 °C. Simultaneously, effectors (NK-92 cells in isolation) were set up as a background control. After 1.5 h, the cells were harvested, stained with fluorescein-conjugated anti-CD56 (NK cell marker, Pacific Blue, Biolegend, San Diego, CA, USA) and anti-CD107a/LAMP1 (Allophycocyanin (APC), Biolegend) antibodies. The cells were then analyzed using flow cytometry as previously described [11] using FlowJo 10.2 software (Ashland, OR, USA).

### 2.5. NK-92 Cell Cytotoxicity Assays

FV-infected NK-92 cells and K562 target cells were mixed together at a 5:1 ratio and incubated in 5% CO_2_ at 37 °C for 4 h. Simultaneously, K562 cells in the absence of NK-92 effector cells were set up as a background control. The cells were harvested and stained with fluorescein-conjugated anti-CD56 and live/dead fixable cell dead reagent (Invitrogen, Waltham, MA, USA). The cells were then analyzed for cell death by flow cytometry as previously described [11].

## 3. Results

From 17 April to 9 July 2020, 45 children with suspected MIS-C were admitted to the Northwell Cohen Children’s Medical Center. A total of 39 patients (86.6%) met the CDC definition for MIS-C (Table 1). The remaining six patients had symptoms suggestive of MIS-C but either did not meet the CDC definition or were found to have another etiology for their presentation. No gene variants were identified among these patients, and they served as similarly sick contemporary controls.

Fifty-four rare (novel or prevalence of <0.1%) VUS were identified in the immune regulatory genes screened across the MIS-C cohort (Supplemental Table S2). Twenty-nine of twenty-nine MIS-C patients (74.3%) had at least one genetic variant. Rare DOCK8 gene missense (amino acid change) mutations were the most prevalent variants, as they were identified in four patients (10.2%). In addition, there were six patients (15.3%) with rare heterozygous missense mutations identified in genes associated with pHLH [12] (LYST in two patients; STXBP2, UNC13D, PRF1, and AP3B1 in one each). Another patient had a missense variant in NLRC4, an autoinflammatory gene recently reported to result in CSS in infants with various dominant missense mutations [13]. Although variants in several immune function genes were identified in the control group, no variants in any of the pHLH genes or DOCK8 were identified in the control group as compared to 25% of children with MIS-C having one of those variants.

Table 1 shows the clinical characteristics of the different patient groups. The 45 children who were initially screened are divided into four groups: Group 1 consisted of 39 children who met criteria for MIS-C. Group 2 consisted of six children who did not meet MIS-C criteria. Out of the 39 children with MIS-C in Group 1, 10 children (Group 1a) had no VUS, 19 children (Group 1b) had a VUS other than DOCK8/pHLH, and 10 children (Group 1c) had a VUS either in the DOCK 8 gene or one of the other pHLH genes. Group 1c.1 is a subset of Group 1c describing 4 out of the 10 children who have a DOCK8 VUS. There was no statistically significant demographic, clinical, or laboratory difference among the groups.

The mean age of the 39 MIS-C patients was 8.97 years (range 2–18 years) with a male predominance (61.5%). The mean length of hospital stay was 6.7 days (range 2–18 days) with 64.1% of patients requiring intensive care. Clinically, this MIS-C cohort was similar to other groups previously reported in the United States [5,14,15] (Table 1). Sixty-four percent of children with MIS-C were admitted to the pediatric intensive care unit (PICU), while forty-nine percent of children needed oxygen. However, none of the children in the control group were admitted to the PICU or required oxygen. There was a trend of increased serum ferritin levels and platelet counts in the group of children with non- DOCK8/pHLH gene variants when compared to the children with those variants. However, the differences did not achieve statistical significance (*p* = 0.45 and *p* = 0.37, respectively, using the Mann–Whitney U test).

**Figure 1:**
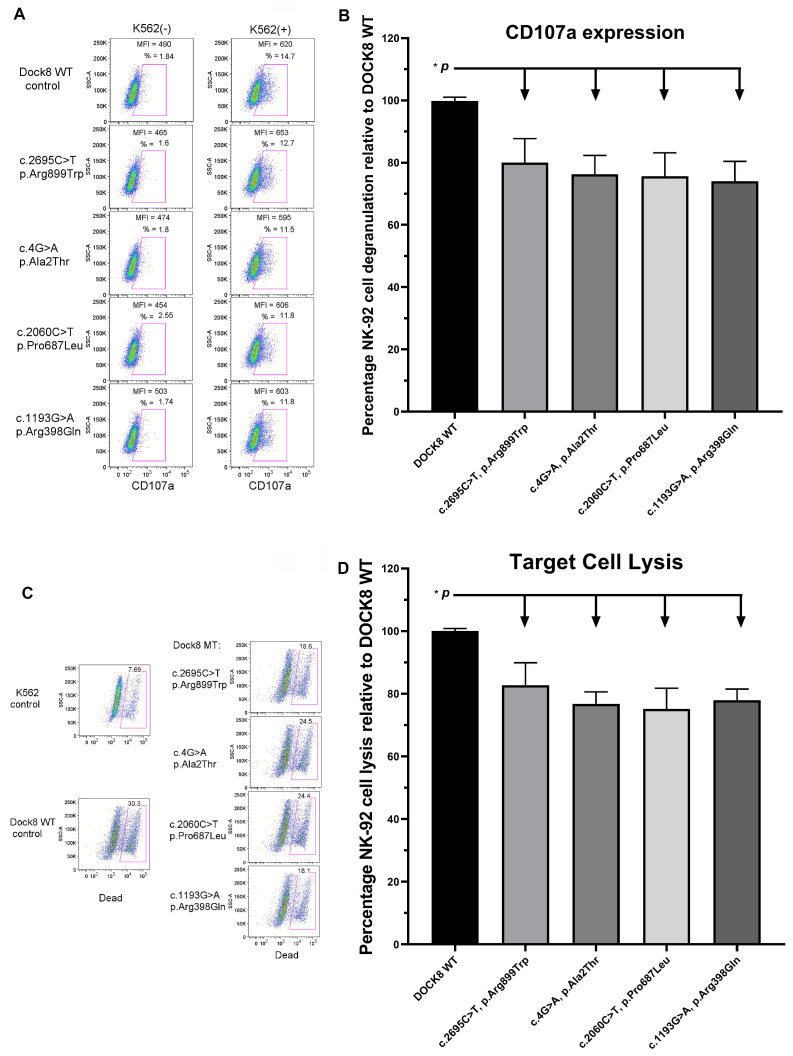
DOCK8 missense mutations from MIS-C patients are found to decrease NK cell degranulation and cytotoxicity. (**A**). Flow cytometry pseudo-color plots reveal decreased CD107a expression (degranulation) in FV-transfected NK-92 cells that express the 4 different patient-derived DOCK8 mutations in comparison with WT DOCK8-transfected NK-92 (control). (**B**). Summary bar graph shows CD107a expression levels in FV-transfected NK-92 cells that express 4 different patient-derived DOCK8 mutations relative to WT DOCK8-transfected cells. Statistical analysis shows (*n* = 4, means ± SD %) significant differences (*t* test comparisons between WT and each mutant, *p* < 0.05 for each). (**C**). Flow cytometry pseudo-color plots reveal decreased cytotoxicity in FV-transfected NK-92 cells that express the 4 different patient-derived DOCK8 mutations in comparison with WT DOCK8-transfected NK-92 (control). (**D**). Summary bar graph shows percentage cytotoxicity values in FV-transfected NK-92 cells that express 4 different patient-derived DOCK8 mutations relative to WT DOCK8-transfected cells. Statistical analysis shows (*n* = 4, means ± SD %) significant differences (denoted by *, *t* test comparisons between WT and each mutant, *p* < 0.05 for each).

By using FV gene transfer into human NK-92 cells, we observed that the four DOCK8 missense variants led to decreased NK cell degranulation and cytotoxicity compared to WT DOCK8 in vitro (Figure 1). Following activation with K562 target cells, CD107a expression levels on NK-92 cells expressing the observed DOCK8 variants were significantly lower than those transduced with WT DOCK8 (*t* test comparison between WT and each mutant, *p* < 0.05 for each; Figure 1A,B). Similarly, the NK-92 cells expressing DOCK8 variants demonstrated decreased cytotoxicity against K562 target cells (*t* test comparison between WT and each mutant, *p* < 0.05 for each; Figure 1C,D).

## 4. Discussion

Infection with SARS-CoV-2 is mild in the overwhelming majority of children when compared to adults. However, despite this mild or asymptomatic initial infection, some children develop hyperinflammatory MIS-C after a 3-to-8-week lag period. The clinical features, cytokine profile, lower naïve CD4 T cell numbers, and presence of autoantibodies place MIS-C at the intersection of severe COVID-19 infection and Kawasaki disease [16,17]. That only some SARS-CoV-2-infected children develop MIS-C (<1 out of 1000 infected) and most do not suggests the possibility of genetic predisposition.

Although there are key differences between MIS-C, CSS, and pHLH, they share the phenomenon of fever, hypercytokinemia, increased markers of inflammation, multiorgan failure, and a temporal association with an acute viral infection. Along these lines, reports have identified SARS-CoV-2 as a trigger for pHLH and secondary HLH [18,19]. Recently, a child with pHLH (STX11 homozygous mutations) was reported to have MIS-C following SARS-CoV-2 infection [19]. Another report also identified some MIS-C patients with genetic variants in the XIAP (fHLH) or CYBB immune-related genes [20]. All these cases indicate a possible association between MIS-C and key variants in immune-related genes.

While pHLH results from homozygous mutations in predominantly autosomal recessive genes (e.g., STX11), certain heterozygous hypomorphic or missense dominant-negative mutations in these same genes (e.g., PRF1 [21,22], STXBP2 [23,24], UNC13D [25] RAB27A) [11] have been reported to contribute to CSS, [26] often in combination with an inciting viral infection, via a threshold model of disease [27]. A prior respiratory pandemic pathogen, H1N1 influenza, is one such example, where heterozygous missense mutations in primary HLH genes (PRF1, LYST) were identified in 36% of fatal cases [21]. Similarly, rare heterozygous missense variants in genes which cause pHLH- and HLH-associated DOCK8 mutations [14,28,29] were found in 25.4% of SARS-CoV-2-triggered MIS-C patients reported in our cohort.

The Dedicator of Cytokinesis 8 (DOCK8) gene encodes a protein responsible for the function, structural integrity, and movement of several immune cells, especially T cells and NK cells. The DOCK8 protein is a GTPase which plays a key role in the signaling pathways for cytoskeletal function through actin polymerization. In addition to its role in actin polymerization, DOCK8 affects several other immune functions including the functioning of immune cells. Patients with DOCK8 deficiency have a combined immunodeficiency including a susceptibility to virus-induced malignancies [29]. DOCK8 is required for optimal NK cell lytic activity, similar to pHLH gene mutations [29,30]. DOCK8 homozygous deficiency is associated with Hyper-IgE syndrome, and in vitro studies demonstrate the importance of DOCK8 to NK cell lytic function [29]. Deficiency in the GTPase DOCK8 contributes to decreased NK cell function by hindering F- actin-mediated cytolytic granule polarization to the immunologic synapse [31]. Interestingly, DOCK8 is a binding partner to CDC42 [32], a recently described CSS-associated gene [33].

Our functional data demonstrate that the DOCK8 genetic missense variants that were identified in our cohort led to a functional decrease in NK cell activity in vitro. Similar effects have been observed with other DOCK8 missense mutation sequences introduced into human NK-92 NK cells [30]. In other patients with non-MIS-C-related CSS, DOCK8 missense mutations were shown not only to cause a decrease in the NK cell lytic activity but also an increased expression of IFNy and TNF by >200% [30]. This fits with the demonstration that decreased perforin-medicated cytolysis by NK cells and cytotoxic CD8 T cells results in pathologically prolonged engagement between the defective lymphocyte and its target cell, yielding increased proinflammatory cytokines [11,34,35]. This excess in proinflammatory cytokines is believed to contribute to the multiorgan system failure seen in CSS [36].

Studies identifying individuals with HLH without a pathogenic mutation found in targeted testing have noted other genetic alterations in whole-exome sequencing that would explain molecular defects in immune regulation [30]. Therefore, rare heterozygous missense variants in DOCK8 and pHLH genes (PRF1, UNC13D, STXBP2, AP3B1, and LYST) in our MIS-C cohort may have contributed to the CSS pathology of these patients in a threshold model of disease, where a combination of severe infection and a baseline genetic partial defect in perforin-mediated cytolysis result in excess proinflammatory cytokine production [28]. Heterozygous pathogenic missense mutations in pHLH or DOCK8 may thus contribute to MIS-C pathology via a partial dominant-negative fashion [37].

There are some limitations of this study. While the control group is in some ways ideal for having a similar clinical and temporal presentation, it is limited by its small size, making definitive comparisons difficult. In addition, the commercial genetic panel is not a comprehensive panel. For example, it did not include the SOCS1/3 genes, which have been implicated as intrinsic virulence factors for SARS-CoV2 [38], and a heterozygous loss of function resulting from SOCS1 mutation was also reported in one patient with MIS-C [39]. Another potential drawback is the fact that these rare VUS identified in this cohort, except the DOCK8 mutations, have not been studied functionally. Nevertheless, we report this observation to highlight clustering of variants in these immunoregulatory genes, particularly those associated with HLH, in a cohort of patients with MIS-C. Perhaps further genetic, functional, and molecular studies will increase our understanding of the pathophysiology of MIS-C. There is an ongoing prospective trial to identify the immune signature in children with MIS-C (ClinicalTrials.gov Identifier: NCT04588363). Several larger studies looking at genetic data in adults with COVID-19 infection to find a genotype–phenotype correlation are underway (the COVID human genome project, https://www.covidhge.com/, accessed on 28 January 2022). Similar studies in children with MIS-C are urgently needed to better understand this unusual immune dysregulation in a subset of children with a delayed inflammatory response to an acute viral infection for early identification and institution of timely management strategies.

## 5. Conclusions

Rare missense heterozygous variants of DOCK8 and other pHLH genes were observed in our cohort of children with MIS-C. Functional data showing the deleterious effects of DOCK8 variants on NK cell function point to a possible role of these variants in the pathophysiology of MIS-C. Larger genomic studies are needed in children with MIS-C to evaluate the role of genetic variants as a pathogenic risk factor for MIS-C development.

## Figures and Tables

**Table 1:** Clinical characteristics of MIS-C patients and controls with and without immune dysfunction gene mutations.

Parameter (Mean/SD)	GROUP 1 MIS-C Group	GROUP 2 Non-MIS-C Group	GROUP 1a MIS-C with No VUS	GROUP 1b MIS-C with a VUS Other Than DOCK8/pHLH	GROUP 1c MIS-C with Either DOCK8 or pHLH VUS	GROUP 1c.1 (Subgroup of 1c) MIS-C with Only DOCK8 VUS
No. of patients	*n* = 39	*n* = 6	*n* = 10	*n* = 19	*n* = 10	*n* = 4
Age (yrs)	9.0 ± 4.2	5.5 ± 3.1	10.5 ± 3.1	9.19 ± 4.42	7.4 ± 4.5	8.0 ± 6.3
Gender	Male 62%	Male 66.7%	Male 55.6%	Male 66%	Male 60%	Male 75%
Presenting symptoms	Fever (97%) Abd. pain (72%) Rash (53%)	Fever (100%) Abd. pain (65%) Rash (40.2%)	Fever (100%) Abd. pain (55.6%) Rash (55.6%)	Fever (95%) Abd. pain (81%) Rash (57.2%)	Fever (100%) Abd. pain (71%) Rash (42%)	Fever (100%) Abd. pain/Rash (both 33%)
Length of stay (days)	6.7 ± 3.7	4.0 ± 3.0	5.4 ± 2.7	7.48 ± 4.26	5.6 ± 2.4	6.7 ± 3.5
PICU stay	Yes (64%)	No (100%)	Yes (77.8%)	Yes (61.9%)	Yes (50%)	Yes (75%)
Oxygen	Yes (49%)	No (100%)	Yes (55.6%)	Yes (52.4%)	Yes (50%)	Yes (75%)
WBC (×10^3^/dL)	11.0 ± 6.3	12.3 ± 5.8	10.27 ± 5.01	11.8 ± 7.7	9.4 ± 3.8	8.6 ± 1.9
Hb (g/dL)	11.2 ± 1.5	10.78 ± 1.5	11.08 ± 1.58	11.4 ± 1.5	10.7 ± 1.1	11.0 ± 1.8
Plt (×10^3^/dL)	187 ± 80	163 ± 73	170 ± 48	204 ± 96	159 ± 51	154 ± 45
Fibrinogen	787 ± 203	509 ± 150	768 ± 216	799 ± 200	756 ± 211	665 ± 149
D-dimer	2137 ± 1652	915 ± 828	2712 ± 1455	1810 ± 1684	2090 ± 1757	2363 ± 1544
LDH	351 ± 136	315 ± 39	335 ± 172	366 ± 136	330 ± 86	230 ± 15
Ferritin	972 ± 1177	447 ± 515	1318 ± 1520	958.44 ± 1178	531 ± 440	864 ± 614
Procalcitonin	11.3 ± 19.3	29 ± 24.2	10.67 ± 11.9	10.1 ± 24	11.8 ± 11.5	9.8 ± 5.3
CRP	191 ± 107	95 ± 52	236 ± 90	185.1 ± 123.7	150 ± 75	166 ± 98
Echocardiography findings	WNL (30%) Cor. ch. (22%) Vent. dys. (10%) Valv. dys. (35%)	WNL (66.6%) Hypokinesia (33.3%)	WNL (22.2%) Hypokinesia (33.3%)	WNL (30%) Cor. ch. (25%) Vent. dys. (10%) Valv. dys. (40%)	WNL (56%) Cor. ch. (33%) Vent. dys. (11%) Valv. dys. (22%)	WNL (50%) Cor. ch. (50%)

MIS-C, multisystem inflammatory syndrome in children; PICU, pediatric intensive care unit; WBC, white blood cells; Hb, hemoglobin; Plt, platelet count; LDH, lactate dehydrogenase; CRP, C-reactive protein; pHLH, primary hemophagocytic lymphohistiocytosis; yrs, years; Abd., abdominal; Cor. ch., coronary changes; Valv. dys., valve dysfunction; Vent. dys., ventricle dysfunction.

## Data Availability

The data that support the findings of this study are available from the corresponding author upon reasonable request.

## Supplementary Materials

The following supporting information can be downloaded at: https://www.mdpi.com/article/10.3390/biology11030417/s1, Table S1: The 109-immune-gene panel commercially screened for exome mutations. Table S2: All immune dysfunction gene variants of unknown significance in patients with MIS-C.
