# Supplementary material for: Hemophagocytic Lymphohistiocytosis Gene Variants in Multisystem Inflammatory Syndrome in Children
Source: Biology (Basel). 2022 Mar 9;11(3):417. doi: 10.3390/biology11030417 (PMC8945334; doi:10.3390/biology11030417)
Supplement: Supplementary file 1 [file biology-11-00417-s001.zip › Supplementary Table S1.pdf]

Table S1. The 109 immune gene panel commercially screened for exome mutations

ACP5	IL21	RAG2
ADA	IL21R	RBCK1
ADA2	IL2RA	RFX5
ADAM17	IL2RG	RFXANK
ADAR	IL36RN	RFXAP
AICDA	ITCH	RNASEH2A
AIRE	ITGB2	RNASEH2B
AP3B1	ITK	RNASEH2C
BLOC1S6	LIG4	RTEL1
BTK	LPIN2	SAMHD1
CARD14	LRBA	SH2D1A
CASP10	LYST	SH3BP2
CASP8	MAGT1	SLC37A4
CD27	MEFV	SLC7A7
CD3G	MVK	STAT1
CD40LG	NCF2	STAT3
COPA	NCF4	STAT5B
CR2	NFAT5	STIM1
CTLA4	NFKB2	STX11
CYBA	NFKBIA	STXBP2
CYBB	NLRC4	TBX1
DCLRE1C	NLRP12	TMEM173
DKC1	NLRP3	TNFRSF13B
DOCK8	NOD2	TNFRSF13C
ELANE	ORAI1	TNFRSF1A
FADD	PIK3CD	TNFSF12
FAS	PIK3R1	TPP2
FASLG	PLCG2	TREX1
FOXP3	PNP	TRNT1
G6PC3	PRF1	TTC7A
ICOS	PRKCD	UNC13D
IFIH1	PSMB8	UNG
IL10	PSTPIP1	WAS
IL10RA	RAB27A	XIAP
IL10RB	RAC2	ZAP70
IL1RN	RAG1	
