# Supplementary material for: Hemophagocytic Lymphohistiocytosis Gene Variants in Multisystem Inflammatory Syndrome in Children
Source: Biology (Basel). 2022 Mar 9;11(3):417. doi: 10.3390/biology11030417 (PMC8945334; doi:10.3390/biology11030417)
Supplement: Supplementary file 1 [file biology-11-00417-s001.zip › Supplementary Table S2.pdf]

Table S2. All immune dysfunction gene variants of unknown significance in patients with MIS-C.

ID	Age	Gender	Race	Variants of unknown significance	Variant population frequency (%)
1	17	M	SE Asian	TPP2 c.1732T>C (p.Ser578Pro)	0.1
2	8	F	Hispanic	CR2 c.1628T>C (p.Leu543Ser);<br>SLC29A3 c.799G>A (p.Ala267Thr)	NP;<br>NP
3	4	M	Hispanic	DOCK8 c.2695C>T (p.Arg899Trp);<br>TBX1 c.1055C>T (p.Pro352Leu)	0.03;<br>UR
4	18	M	Greek	RAG1 c.193G>A (p.Val65Ile)	NP
5	15	M	Af. Am.	CARD14 c.623C>A (p.Ala208Glu)	NP
6	3	F	Af. Am.	STXBP2 c.1772C>A (p.Ala591Asp)	0.2
7	10	M	SE Asian	RAG1 c.1321G>T (p.Ala441Ser)	NP
8	8	M	Af. Am.	DCLRE1C c.1340C>T (p.Thr447Ile);<br>STAT1 c.1591G>A (p.Ala531Thr)	NP;<br>0.1
9	8	M	Hispanic	TNFRSF1A c.596T>C (p.Ile199Thr);<br>ADAR c.533C>T (p.Ser178Phe)	0.008;<br>NP
10	4	M	Af. Am.	LRBA c.3407C>T (p.Pro1136Leu);<br>LRBA c.4930C>G (p.Leu1644Val);<br>LRBA c.7828A>G (p.Ile2610Val);<br>PIK3CD c.455C>T (p.Ala152Val)	0.07;<br>0.06;<br>NP;<br>UR
11	8	F	Hispanic	DCLRE1C c.1733A>G (p.Tyr578Cys);<br>LYST c.2030T>C (p.Ile677Thr)	0.04;<br>NP
12	5	F	Af. Am.	IFIH1 c.1793G>A (p.Arg598His)	0.05
13	5	M	Af. Am.	NOD2 c.380C>T (p.Ser127Leu)	0.05
14	7	M	Hispanic	ADA2 c.1385T>C (p.Ile462Thr);<br>ADA2 c.1474A>G (p.Thr492Ala)	0.1;<br>0.04
15	10	M	Hispanic	IL1RN c.258G>A (Silent);<br>TNFRSF13B c.178C>T (p.Arg60Cys)	NP;<br>0.009
16	4	M	Hispanic	ADA c.145G>A (p.Val49Ile)	NP
17	2	M	Caucasian	CR2 c.3258T>G (p.Asp1086Glu);<br>ZAP70 c.1153C>T (p.Arg385Cys)	NP;<br>0.01
18	10	M	Hispanic	DOCK8 c.2060C>T (p.Pro687Leu)	0.04
19	9	M	SE Asian	NLRC4 c.1147G>C (p.Ala383Pro);<br>NOD2 c.1390G>T (p.Gly464Trp)	NP;<br>0.08
20	7	M	Af. Am.	PLCG2 c.2231A>C (p.Asn744Thr);<br>LPIN2 c.1133C>T (p.Pro378Leu);<br>LRBA c.6703C>T (p.Arg2235Trp);<br>LYST c.10669G>T (p.Val3557Leu);<br>SLC29A3 c.1202G>A (p.Arg401His);<br>TPP2 c.3526C>G (p.Leu1176Val)	NP;<br>0.07;<br>0.01;<br>0.1;<br>0.01;<br>0.01
21	7	F	Other	ADAR c.931T>A (p.Ser311Thr);<br>TMEM173 c.575G>T (p.Gly192Val)	NP;<br>0.09
22	8	F	Af. Am.	CARD14 c.2956C>T (p.Arg986Cys)	0.1
23	6	F	Hispanic	IFIH1 c.467A>C (p.Glu156Ala);<br>ORAI1 c.763G>A (p.Val255Ile)	0.04<br>0.06
24	13	M	Multi	NCF2 c.551G>A (p.Arg184Gln)	NP
25	9	F	Af. Am.	RMRP n.50C>T (RNA change)	UR

26	2	M	Af. Am.	LRBA c.4918G>A (p.Val1640Met);<br>LRBA c.1161+4G>T (Intronic);<br>PSTPIP1 c.469G>A (p.Ala157Thr);<br>DOCK8 c.1193G>A (p.Arg398Gln);<br>RAG1 c.2237T>G (p.Phe746Cys)	0.07;<br>0.06;<br>NP;<br>0.08;<br>0.01
27	8	M	Guyanese	SH3BP2 c.1205T>G (p.Leu402Arg)<br>UNC13D c.796C>T (p.Arg266Cys)	NP;<br>0.02
28	14	F	Guyanese	ACP5 c.619C>A (p.Pro207Thr);<br>CR2 c.1282C>T (p.Arg428Cys);<br>IL10RA c.700A>G (p.Thr234Ala);<br>PRF1 c.1424G>A (p.Gly475Glu)	NP;<br>0.1;<br>0.05;<br>0.006
29	14	F	Af. Am.	AP3B1 c.1862C>G (p.Thr621Ser) ;<br>DOCK8 c.4G>A (p.Ala2Thr) ;<br>TPP2 c.340A>G (p.Ile114Val) ;<br>TTC74 c.826A>T (p.Thr276Ser)	NP;<br>NP;<br>0.003;<br>NP

NP, Not present in population database; UR, Variant frequency unreliable; SE, Southeast; Af. Am.; African American; Multi, multiracial; M, male; F, female.
